# The Effectiveness of Cognitive Bias Modification Interventions for Substance Addictions: A Meta-Analysis

**DOI:** 10.1371/journal.pone.0162226

**Published:** 2016-09-09

**Authors:** Ioana A. Cristea, Robin N. Kok, Pim Cuijpers

**Affiliations:** 1 Department of Clinical Psychology and Psychotherapy, Babeş-Bolyai University, Cluj-Napoca, Romania; 2 Department of General Psychology, University of Padova, Padova, Italy; 3 Department of Psychology, Faculty of Health Sciences, University of Southern Denmark, Odense, Denmark; 4 Centre for Innovative Medical Technology, Department of Clinical Innovation, Odense University Hospital, Odense, Denmark; 5 Department of Clinical Psychology, Faculty of Behavioural and Movement Sciences, VU University Amsterdam, Amsterdam, The Netherlands; 6 EMGO Institute for Health and Care Research, VU University and VU University Medical Centre, Amsterdam, the Netherlands; Radboud Universiteit, NETHERLANDS

## Abstract

**Background and Aims:**

Cognitive bias modification (CBM) interventions, presumably targeting automatic processes, are considered particularly promising for addictions. We conducted a meta-analysis examining randomized controlled trials (RCTs) of CBM for substance addiction outcomes.

**Methods:**

Studies were identified through systematic searches in bibliographical databases. We included RCTs of CBM interventions, alone or in combination with other treatments, for any type of addiction. We examined trial risk of bias, publication bias and possible moderators. Effects sizes were computed for post-test and follow-up, using a random-effects model. We grouped outcome measures and reported results for addiction (all related measures), craving and cognitive bias.

**Results:**

We identified 25 trials, 18 for alcohol problems, and 7 for smoking. At post-test, there was no significant effect of CBM for addiction, *g* = 0.08 (95% CI -0.02 to 0.18) or craving, *g* = 0.05 (95% CI -0.06 to 0.16), but there was a significant, moderate effect on cognitive bias, *g* = 0.60 (95% CI 0.39 to 0.79). Results were similar for alcohol and smoking outcomes taken separately. Follow-up addiction outcomes were reported in 7 trials, resulting in a small but significant effect of CBM, *g* = 0.18 (95% CI 0.03 to 0.32). Results for addiction and craving did not differ by substance type, sample type, delivery setting, bias targeted or number of sessions. Risk of bias was high or uncertain in most trials, for most criteria considered. Meta-regression analyses revealed significant inverse relationships between risk of bias and effect sizes for addiction outcomes and craving. The relationship between cognitive bias and respectively addiction ESs was not significant. There was consistent evidence of publication bias in the form of funnel plot asymmetry.

**Conclusions:**

Our results cast serious doubts on the clinical utility of CBM interventions for addiction problems, but sounder methodological trials are necessary before this issue can be settled. We found no indication that positive effects on biases translate into effects on addiction outcomes.

## Background

Despite mounting enthusiasm for cognitive bias modification over the last decade, expressed in an explosion of studies and research programs [1], as well as highly laudatory reviews and dedicated comments in top-tier journals [2,3] and exclusively positive coverage in the popular media [4], the clinical effectiveness of these bias modification interventions has recently been contested for anxiety and depression related outcomes, in both adult [5,6] and young populations [7]. Nonetheless, one of the fields where CBM is still considered as particularly promising is that of addiction and substance use disorders, where studies have been accumulating during the last years.

Cognitive bias modification (CBM) has been defined synthetically as the “direct manipulation of a target cognitive bias, by extended exposure to task contingencies that favor predetermined patterns of processing selectivity” [8] (p.191). CBM interventions are grafted on a dual process model of addiction, which postulates that relatively automatic (impulsive) processes surmount controlled (reflective) ones (see [9] for a review). In this framework, CBM interventions are believed to effectively impact the impulsive processes, considered less amendable to change within traditional psychological interventions. Two types of automatic processes in particular have been studied. The first refers to attentional biases, the preferential allocation of attention resources to substance-related stimuli [10]. The second concentrates on presumably automatically activated action tendencies of approaching addiction-related stimuli [9]. A number of procedures were developed to target each type of bias, mostly based on experimental, computer-based tasks in which participants are trained, over more repetitions of pairs of stimuli, and usually without their explicit awareness, to either avoid directing attention to addiction-related stimuli (attention bias modification/ABM) or to avoid approaching them (approach-avoidance task/AAT).

In a recent review, Cox and colleagues [10] argued that ABM should be awarded “more weight” for addiction disorders and that the preliminary evidence about its effectiveness, available so far, was “encouraging” (p.222). AAT was also judged as efficacious for reducing alcohol use [11] and alcohol dependence [9]. Although a recent narrative review has voiced concerns about the clinical potential of ABM in substance use disorders, drawing attention to methodological flaws and numerous negative findings [12], a systematic review examining the effectiveness of these interventions for addiction has not yet been conducted. Consequently, our goal was to conduct a meta-analysis examining all types of CBM interventions tested in randomized controlled trials (RCTs) for addiction-related outcomes.

## Methods

### Identification and selection of studies

We conducted a comprehensive literature search (see S1 File. for the complete search string) in Pubmed, PsychInfo, the Cochrane library and EMBASE through March 2015. The search was then updated on December 8^th^ 2015, looking for studies published between March and December. We combined text words related to CBM interventions, ("cognitive bias", "attention* bias", "interpret* bias" or "approach avoidance") and ("modification", "training", "practice", or "task") with terms relating to addictions, ("addiction", "dependen*", "alcohol", "drinking", "tobacco", "nicotine”, "smoking", "drug", "cannabis", "marijuana", "cocaine", "heroin", "opiates", "amphetamine" or "substance *use"). No MeSH terms were available. No filters were used as to not miss any studies that might not have presented themselves as RCTs, but as experimental studies. The reference sections of previous reviews were also checked for additional articles. There was no registered protocol for this systematic review.

Included studies were: (a) randomized controlled trials (RCTs), comparing (b) a CBM intervention, alone or in combination with another treatment (c) to a control group, another active treatment, or a combination of treatments, (d) for addiction relevant outcomes, (e) published in peer-reviewed journals. We did not apply any age or language restrictions.

We included studies employing a combination of a CBM intervention with another active treatment (psychological or pharmacological), if there was a control condition for the CBM intervention (i.e. a group that received no CBM intervention to decrease bias, whether that meant no intervention, a placebo / no-contingency intervention, an intervention supposed to achieve the reverse effect of increasing bias), whether it was alone or combined with another active treatment. Simply put, the CBM intervention had to be different between the intervention and control groups.

Active CBM interventions were defined as intending to *decrease* bias, regardless of its type and consequently improve addiction relevant outcomes. Using only outcomes measured on established, standardized instruments would have been preferable, but for craving many studies only reported outcomes on *ad-hoc* Likert-type or Visual Analogue Scales (VAS). Therefore, we included these measures so as not to lose too many studies from the analyses, but conducted sensitivity analyses to verify whether results changed when exclusively considering established and standardized measures. We did not include outcomes that measured consumption or preferences during a non-standardized behavioral test (i.e., participants were given an impromptu taste test which measured whether they chose non-alcoholic over alcoholic drinks or how much they consumed from each drink). We opted for this because this is a non-standardized and variable task that does not take into account participants’ general preferences, their habitual alcohol consumption, and that is usually carried out without their awareness. We did include measures of consumption when they were reported on standardized self-report (e.g., The Timeline Follow-Back diary) or behavioral measures.

### Power calculation

Based on previous narrative reviews and our own previous meta-analysis of CBM [7], we expected a reduced number of studies and a small effect size. We conducted a power calculation [13] to determine how many studies would have to be included to ensure enough statistical power to identify relevant effects. We conservatively assumed a medium level of between-study variance, τ^2^, a statistical power of 0.80, and a significance level, alpha, of 0.05. These calculations indicated that it would take at least 20 studies with a mean sample size of 30 (15 participants per condition) to be able to detect a small effect size of d = 0.30. Alternatively, we would need to include 15 studies with a total mean number of 40 participants each to detect an effect size of d = 0.30, or 14 studies with 50 participants.

### Risk of bias (RoB) assessment and data extraction

As a proxy for methodological quality, we used five criteria of the Cochrane Collaborations’s *Risk of Bias* tool [14], developed to assess sources of bias in RCTs:

1. Adequate generation of allocation sequence
2. Concealment of allocation to conditions
3. Prevention of knowledge of the allocated intervention to assessors of outcome
4. Prevention of knowledge of the allocated intervention to participants
5. Dealing with incomplete data

Blinding of outcome assessors was rated as low risk of bias either if the study described proper methods of ensuring blinding or if all outcome measures were self-report scales, thus not requiring the interaction with an assessor. We also evaluated blinding of participants because, unlike other psychological interventions, CBM is often carried out without making participants aware of the purpose of the intervention, the particular contingency they are exposed to, and in some cases, to the very fact they are being subjects of an intervention. Moreover, lack of participant awareness of the training contingencies was interpreted as evidence of the implicit mechanism of action behind CBM interventions, believed to modify biases inaccessible to awareness [1,15]. We rated studies as having low risk of bias on this criterion only if the authors explicitly measured participant awareness of training contingencies and reported that less than 50% of the study participants were aware of their group allocation.

Dealing with incomplete data was rated as low risk of bias if there was no missing data or if authors explicitly reported analyzing their data using the intent-to-treat (ITT) principle (i.e., *all* randomized participants were analyzed). Risk of bias was rated by two independent researchers (IAC and RNK). Disagreements were discussed and if they remained unresolved, the third author (PC) was consulted. Along with describing risk of bias for the included studies, we also computed a “low risk of bias” score for each study, by awarding one point of each criterion for which the study had low RoB. In this way, we obtained a total score for low risk of bias for each study across the 5 criteria, which we used in meta-regression analyses.

As potential moderators, we extracted information about several aspects of the studies:

1. Addiction type: alcohol or smoking (there were no trials for other substances)
2. Participants: patients- diagnosed using a clinical interview (e.g., Composite International Diagnostic Interview); substance consumers/users- selected for high values on a scale or for self-reported frequent use of a substance
3. Delivery setting: laboratory; home/participant’s environment; mental health clinic
4. Type of bias intervention: attentional (ABM); approach-avoidance (AAT); response inhibition
5. Number of sessions: as many studies had one session, we also coded this variable categorically into single- and multi-session studies.
6. Type of outcome measure used: established, validated standardized instruments or tasks; *ad-hoc* scales (Likert scales, Visual analogue scales); bias-related tasks or measures

### Meta-analysis

Effect sizes (ESs) were computed for each comparison between a CBM intervention (alone or in combination) and a comparison group as the difference between the 2 groups at posttest and, respectively, follow-up (Cohen’s *d* or standardized mean difference). The ESs were calculated by subtracting the mean score of the CBM group from the mean score of the comparison group, and dividing the result by the pooled standard deviation of the two groups. It is generally accepted [16] that ESs of 0.2 are small, while ESs of 0.5, moderate and ESs of 0.8 and over, large. Since many studies had small sample sizes, we reported the indicator corrected for small sample bias [17], Hedges’ *g*.

We used the Comprehensive Meta-analysis software (CMA; version 2.2.064) for computing and pooling individual effect sizes. As there was a lot of variability in the addiction outcomes considered and the instruments used to measure them, we defined the following outcome categories: addiction (all outcomes related to addiction, regardless of the problem type, like habitual use or desire, or the particular substance); and craving (all outcomes relating to craving or desire for a substance, regardless of the substance). We also considered another category related to bias outcomes, where we included all bias measures, regardless of the task or type (avoid/attend or avoid/approach), as it is not clear which type of bias CBM interventions are supposed to influence (i.e., increase avoidance of alcohol related stimuli or decrease attending/approaching of these stimuli).

In the cases where a study used more than one outcome from the same pre-defined category or if the same outcome was measured by more than one instrument, an average ES per study was computed. When means and standard deviations for the relevant outcomes were not reported in a study, we used the procedures implemented in CMA [18] to transform dichotomous data into the standardized mean difference, or other statistics such as t-values or exact p-values to calculate the standardized mean difference. If there was insufficient data for ES calculation, we contacted the authors asking for their data. If more versions of CBM were used in a study, these were averaged and sensitivity analyses were further conducted, using only one ES per study (i.e., the one with the largest ES, and the one with the lowest). For studies with more than one control group for the CBM intervention, we used only one control group to calculate ES. We chose the group most similar to a placebo group (i.e., no contingency training), as this was the most common control condition in CBM studies. This approach also reduces effect size inflation attributable to non-specific effects of the intervention or to the fact that in some cases the control condition was designed to achieve an opposite effect of increasing bias and symptoms. We also calculated ESs at follow-up, since prevention of relapse is considered a particularly important strong point for CBM in addictions [12, 19].

To render results easier to interpret from a clinical point of view, we transformed the standardized mean difference (Hedges’ *g*) into numbers-needed-to-be-treated (NNT), using the formulae of Kraemer & Kupfer [20]. The NNT represents the number of patients that would have to be treated to generate one additional positive outcome compared to the control group [21].

Given that we expected considerable heterogeneity among studies, we employed a random effects model [18] to calculate mean effect sizes. For the assessment of heterogeneity, we calculated the *I*^*2*^ statistic, indicating heterogeneity in percentages. A value of 0% indicates no observed heterogeneity, whereas values over this refer to increasing heterogeneity, with 25% as a threshold for low, 50% for moderate and 75% or above for high [22]. We calculated 95% confidence intervals (CIs) around *I*^*2*^ [23], using the non-central χ^2^-based approach with the heterogi module for STATA [24]. Outliers were defined as studies in which the 95% CI was outside the 95% CI of the pooled studies (on both sides).

We used a mixed effects model for subgroup analyses. Under this model, studies within subgroups are pooled using the random effect model, while tests for significant differences between subgroups are carried out using a fixed effects model. For continuous moderator variables, we used meta-regression analyses to examine whether there was a significant relationship between each of these variables and the ES.

We assessed publication bias using three different procedures. First, we visually inspected the asymmetry of the funnel plot. In the presence of bias, there will be a higher concentration of studies at the lower right part of the plot [18]. Secondly, we used the Duval-Tweedie trim and fill procedure [25] (as implemented in CMA, version 2.2.064, using a fixed effects model and searching for missing studies left of the mean), which gives an estimate of the ES after the publication bias has been taken into account (adjusted effect size) and also indicates how many studies were imputed to correct for publication bias. Thirdly, we conducted Egger’s test of the intercept to test the symmetry of the funnel plot.

## Results

### Selection and inclusion of studies

**Fig 1:**
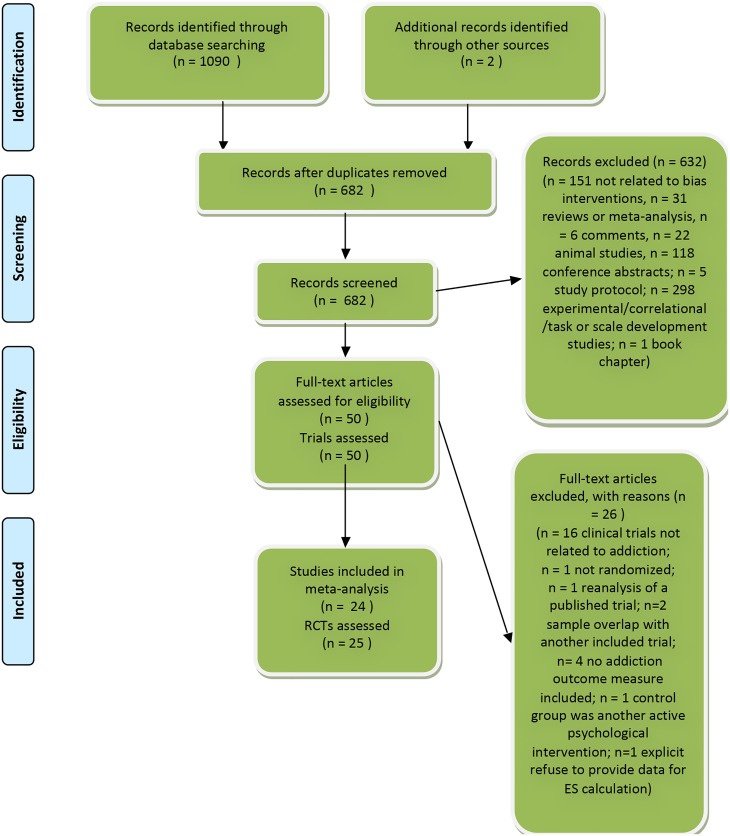
Flowchart of selection and inclusion process, following the PRISMA statement.

We examined a total of 1092 records (682 after duplicates were removed) and excluded 632 based on the inspection of the abstract. We retrieved the full text of the remaining 50 articles. Fig 1 presents the flowchart of the inclusion process and details the reasons for the exclusion of trials, following the PRISMA statement [26]. This process resulted in 24 published studies comprising of 25 RCTs that met our inclusion criteria and were included in the meta-analysis.

### Characteristics of included studies

The 25 RCTs included 34 relevant comparisons (see S2 File. for the complete list of included studies). Most studies were recent, with the earliest one published in 2005, and the bulk of the included studies (11) between 2012 and 2015. Eighteen trials focused on alcohol problems, and 7 on smoking. Twelve studies targeted attentional bias, while 8 targeted approach bias, 4 response inhibition and one focused on interpretation bias. The number of intervention sessions ranged from 1 to 21, but 11 RCTs used only one session. Twenty RCTs focused on consumers of a substance, 5 on clinical patients. Fifteen studies were based on interventions carried out exclusively in the laboratory, 4 at home/the participant’s environment, one gave participants a choice between the laboratory and home, and 5 in a mental health clinic. The follow-up period ranged from 4 weeks to one year, with a mean of 6 months. Table 1 presents selected characteristics of the included RCTs.

**Table 1:** Selected characteristics of included studies of cognitive bias modification interventions.

Study^a^	Population^b^	N_rand_^c^	CBM^d^	Control^e^	Conc Tx^f^	Addiction Measures^g^	Bias measure^h^	D^i^	Ns^j^	FU^k^
Attwood, 2008	Smokers (≥ 5 cig/day)	55	ABM (VPT)	ABM (attend smoking)	-	QSU-B; Craving VAS	VPT	L	1	-
Begh, 2015	Smokers trying to quit (≥10 cig/day)	119	ABM (VPT)	No cont	NP	MPSS-C; MPSS-M; CO-verified abstinence	VPT; PST	C	4	1,2, 3& 6 mths
Boendermaker, 2015 Study 1	Regular drinkers	77	Inhibition (Go/No Go) (original/gamified/social	No cont	-	Alcohol use (TFLB)	Alcohol Go/No Go	L/H	3	-
Cox, 2015	Drinkers (≥14 units/wk-women; ≥21 units/wk-men)	148	AACTP	No training	-	DRQ (MWD; ATWD); SIP	Stroop Task	L	4	3&6 mths
Eberl, 2013	Alcohol dependent inpatients (CIDI)	509	A-AAT	No training	TAU (CBT)	Relapse (DGSS-4)	A-AAT	C	12	1 year
Field, 2005	Drinkers (≥14 units/wk-women; ≥21 units/wk-men)	40	ABM (VPT)	ABM (attend alcohol)	-	DAQ; Urge to drink VAS	VPT	L	1	-
Field, 2007	Drinkers (≥14 units/wk-women; ≥21 units/wk-men)	60	ABM (VPT)	No cont: ABM (attend alcohol)	-	DAQ; Urge to drink VAS	VPT; PST; FICBT; SRCT	L	1	-
Field, 2009	Smokers (≥ 1 cig/wk)	72	ABM (VPT)	No cont; ABM (attend)	-	QSU-B; Urge to smoke VAS	VPT; PST	L	1	-
Houben, 2011	Drinkers (≥10 units/wk-women; ≥12 units/wk-men)	52	Inhibition (Go/No Go)	Go/No Go (alcohol for go)		Alcohol use (TFLB)	IAT	L	1	-
Houben, 2012	Drinkers (≥10 units/wk-women; ≥12 units/wk-men)	57	Inhibition (Go/No Go)	Go/No Go (alcohol for go)	-	Alcohol use (TFLB)	SRCT; SST; IAT	L	1	-
Kerst, 2014	Smokers (≥10 cig/day)	65	ABM (VPT)	No cont	-	QSU; Cig/day (diary); CO (ppm); Cotinine saliva (ng/ml)	VPT	H	21	-
Jones, 2013 St 1	Drinkers (≥14 units/wk-women; ≥21 units/wk-men)	90	Inhibition (SST)	Inhibition (SST) (neutral); Disinhibition (SST no stop)	-	Alcohol use (TFLB); AAAQ	SST	L	1	-
Lindgren, 2015 St 1	Drinkers (1 heavy drink ep≥ 4–5 drinks past mth)	295	A-AAT (original/general ident/personalized ident)	No cont (original/general ident/personalized ident)	-	ACQ-SF-R; Intention to drink	A-AAT	L	2	-
Lindgren, 2015 St 2	Drinkers (AUDIT > 8)	288	A-AAT (original/general ident/personalized ident)	No cont (original/general ident/personalized ident)	-	ACQ-SF-R; Intention to drink	A-AAT	L	2	-
Lopes, 2014	Smokers trying to quit (≥5 cig > 30 days)	67	ABM (VPT)	No cont; ABM+No cont	Group CBT	QSU-B; FTND; Cig/day; CO(ppm)	VPT	L	3	1, 6, & 12 mths
McGeary, 2014	Drinkers (AUDIT > 8)	31	ABM (VPT)	No cont	-	DHQ (1 item)	VPT	L+H	9	-
McHugh, 2010	Smokers (≥10 cig/day for 1 year)	64	ABM (VPT)	No cont	-	QSU-B	VPT	L	1	-
Schoenmakers, 2007	Drinkers (> 20 units/wk; ≥ 1 binge drinking ep in last 2 wks)	106	ABM (VPT)	No cont	-	Craving VAS	VPT; FICBT	L	1	-
Schoenmakers, 2010	Alcohol dependent inpatients (DSM-IV)	43	ABM (VPT)	Categorization task	CBT	DAQ; Relapse	VPT	C	5	3 mths
Wiers CE, 2014	Alcohol dependent inpatients (MINI)	36	A-AAT	No cont	-	DAQ; AAAQ	A-AAT	C	6	-
Wiers RW, 2010	Drinkers (AUDIT > 8)	42	A-AAT	A-AAT (approach)	-	Urge to drink (Likert)	A-AAT	L	1	-
Wiers RW, 2011	Alcohol dependent inpatients (CIDI)	214	A-AAT (explicit/implicit)	No cont; WL	TAU (CBT)	Craving (Likert); Relapse (DGSS-4)	A-AAT	C	4	1 yr
Wiers RW, 2015	Drinkers (AUDIT > 8)	314	A-AAT (100% /90% /explicit 100%); AACTP	No cont	-	Craving VAS; AAAQ; Drinks/day (TFLB)	A-AAT	H	4	1 &2 mths
Wittekind, 2015	Smokers (*ad hoc* online survey)	257	AAT; mAAT	WL	-	CQSS; FTND; CDS-12; OCSS	-	H	1	-
Woud, 2015	Drinkers (AUDIT > 8)	74	CBM-I	CBM-I (alcohol)	-	Alcohol use (TFLB); Urge to drink (Likert)	Similarity ratings	L	3	-

Note.

^a^ St, study

^b^ Cig, cigarette; Wk, Week; Mths, Months; CIDI, Composite International Diagnostic Interview; AUDIT, Alcohol Use Disorders Identification Test; ep, episode; DSM-IV, Diagnostic and Statistic Manual IV; MINI, Mini International Neuropsychiatric Interview;

^c^ N_rand_, Number randomized

^d^ CBM, cognitive bias modification; ABM, attention bias modification; VPT, Visual Probe Task; AACTP, Alcohol Attention Control Training Program; SST, Stop-Signal Task; ident, identity; A-AAT, Alcohol Approach-Avoidance Task; mAAT, modified Approach-Avoidance Task; CBM-I, cognitive bias modification for interpretation;

^e^ ABM, attention bias modification; No cont, No Contingency; SST, Stop-Signal Task; ident, identity; A-AAT, Alcohol Approach-Avoidance Task; WL, Waitlist; CBM-I, cognitive bias modification for interpretation;

^f^ Conc Tx, Concurrent Therapy; NP, Nicotine Patch; TAU, treatment as usual; CBT, cognitive behavior therapy

^g^ QSU-B, Questionnaire of Smoking Urges-Brief; VAS, Visual Analogue Scale; MPSS (C/M), Mood and Physical Symptoms Score (Craving/Mood); DRQ, Drinking Record Questionnaire; MWD, mean weekly drinking; ATWD, atypical weekly drinking; SIP, Short Index of Problems; DGSS-4, German Society for Addiction Research and Adddiction Medicine-4; DAQ, Desires for Alcohol Questionnaire; TFLB, Timeline Follow-Back Questionnaire; CO (ppm),expired alveolar carbon monoxide; AAAQ, Alcohol Approach Avoidance Questionnaire; ACQ-SF-R, Alcohol Craving Questionnaire—Short Form—Revised questionnaire; FTND, Fagerström Test for Nicotine Dependence; DHQ, Drinking Habits Questionnaire; CQSS, Commitment to Quitting Smoking Scale; CDS-12, Cigarette Dependence Scale-12; OCSS, Obsessive Compulsive Smoking Scale

^h^ VPT, Visual Probe Task; A-AAT, Alcohol Approach-Avoidance Task; PST, Pictorial Stroop Task; IAT, Implicit Association Test; FICBT, Flicker-Induced Change Blindness Task; SRCT, Stimulus-Response Compatibility Task; SST, Stop-Signal Task

^i^ D, Delivery; L, Laboratory; H, Home; C, Clinic;

^j^ Ns, Number of sessions;

^k^ FU, Follow-up; Mth, month.

### Risk of bias of the included studies

**Fig 2:**
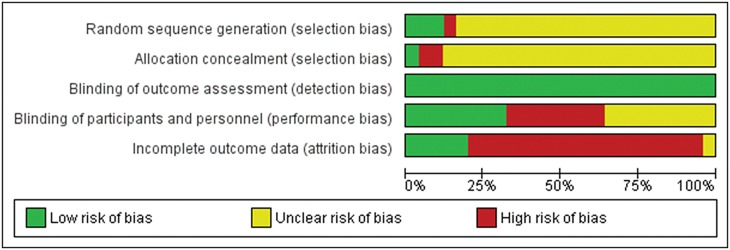
Risk of bias graph: review authors' judgments about each risk of bias item presented as percentages across all included studies.

Overall, most of the included RCTs had high or uncertain risk of bias for most criteria. Only 4 studies out of 25 had low risk of bias for three or more of the criteria considered. Thirteen studies could be rated as low risk of bias on only one of the five criteria. Fig 2 presents the percentage of studies with a low, unclear (i.e., not enough information) and high risk of bias, for each of the quality criteria. For blinding of outcome assessors, 23 trials used exclusively self-report measures for addiction outcomes, and the other 2 employed proper blinding. For sequence generation and allocation concealment, only three and respectively one trial could be rated as having low risk of bias. For blinding of participants, an equal number of studies (8) had low and respectively high RoB. For incomplete outcome data, the vast majority of studies (19/24) had not employed methods to include drop-outs in the analysis. It is also worth mentioning that for all criteria except blinding of assessors and incomplete data, the majority of RCTs did not provide the information necessary for assessing them (21/25 for sequence generation, 22/25 allocation concealment, and 9/25 for blinding of participants). A risk of bias summary for each included trial is presented in S1 Fig.

### CBM, compared to a control condition: Power analysis

We were able to identify 24 RCTs with a mean number of 68 subjects (34 per condition) for post-test outcomes, and 7 RCTs with a mean number of 136 (68 per condition) subjects for follow-up. According to our power analysis, this would allow us to evidence a small ES of 0.30 or more for post-test, but not for follow-up.

### Posttest results- Addiction (all outcomes)

The effects of a CBM intervention were compared to a control condition in 25 RCTs, with 34 comparisons (Table 2). The mean ES was small and non-significant with *g* = 0.08 (95% CI -0.02 to 0.18). Heterogeneity was low (*I*^*2*^ = 0%; 95% CI: 0–40). Results were similar when considering only established outcome measures, and respectively when excluding comparisons where the control group received the opposite intervention (i.e., meant to increase bias). The effects were non-significant in the four studies that employed CBM in addition to another active intervention (physical or psychological), *g* = 0.06, 95% CI -0.16 to 0.28.

**Table 2:** Effects of CBM interventions, compared to control, at posttest and follow-up, for addiction outcomes^a^.

Variable	n	*g*	95% CI	I^2^	I^2^ 95% CI	NNT	p^b^
**Addiction (all measures)**	24	0.08	-0.02 to 0.18	0	0~40	21.74	
One ES per study (only highest)	24	0.11	0.009 to 0.20	0	0~40	16.13	
One ES per study (only lowest)	24	0.05	-0.05 to 0.15	0	0~40	35.71	
Established outcome measures only	21	0.09	-0.02 to 0.20	0	0~41	20	
Comparisons with increase bias interventions excluded^c^	18	0.04	-0.07 to 0.15	0	0~44	45.45	
Studies of CBM combined with another intervention^d^	4	0.06	-0.16 to 0.28	7	0~70	29.41	
*Subgroup analysis*^e^							
Addiction type Alcohol	17	0.10	-0.01 to 0.22	0	0~45	17.86	0.459
Smoking	7	0.02	-0.15 to 0.20	0	0~58	83.33	
Sample type Consumers	20	0.08	-0.03 to 0.19	0	0~42	21.74	0.979
Patients	4	0.07	-0.19 to 0.34	30	0~76	25	
Delivery setting^f^ Laboratory	15	0.07	-0.06 to 0.21	0	0~46	25	0.999
Home	4	0.07	-0.15 to 0.28	0	0~68	25	
Clinic	4	0.07	-0.19 to 0.34	30	0~76	25	
Bias targeted^g^ Approach	7	0.07	-0.08 to 0.23	0	0~58	25	0.437
Attentional	12	0.03	-0.12 to 0.18	0	0~50	62.5	
Inhibition	4	0.23	-0.04 to 0.50	0	0~68	7.69	
Number of sessions Single	11	0.07	-0.07 to 0.21	0	0~51	25	0.867
Multiple	13	0.09	-0.05 to 0.22	0	0~49	20	
**Craving**	18	0.05	-0.06 to 0.16	0	0~44	35.71	
One ES per study (only highest)	18	0.08	-0.03 to 0.20	2	0~44	21.74	
One ES per study (only lowest)	18	0.02	-0.09 to 0.14	0	0~44	83.33	
Established outcome measures only	14	0.05	-0.09 to 0.19	0	0~49	35.71	
Comparisons with increase bias interventions excluded^h^	14	0.02	-0.11 to 0.14	0	0~47	83.33	
Studies of CBM combined with another intervention^d^	4	0.03	-0.32 to 0.38	56	0~83	62.5	
*Subgroup analysis*^e^							
Addiction type Alcohol	12	0.07	-0.07 to 0.20	0	0~50	25	0.798
Smoking	6	0.03	-0.22 to 0.28	27	0~71	62.5	
Sample type Consumers	14	0.05	-0.08 to 0.19	0	0~47	35.71	0.908
Patients	4	0.03	-0.32 to 0.38	56	0~83	62.5	
Delivery setting Laboratory	12	0.06	-0.09 to 0.21	0	0~50	29.41	0.972
Home	2	0.02	-0.35 to 0.39	0	N/A^i^	83.33	
Clinic	4	0.03	-0.32 to 0.38	56	0~83	62.5	
Bias targeted^j^ Approach	6	0.10	-0.07 to 0.28	0	0~61	17.86	0.36
Attentional	10	-0.01	-0.19 to 0.16	11	0~58	166.67	
Number of sessions Single	8	0.04	-0.15 to 0.22	0	0~56	45.45	0.895
Multiple	10	0.05	-0.10 to 0.21	10	0~57	35.71	
**Addiction (all measures)- follow-up**	7	0.18	0.03 to 0.32	0	0~58	9.80	
One ES per study (only highest)	7	0.18	0.04 to 0.32	0	0~58	9.80	
One ES per study (only lowest)	7	0.16	0.02 to 0.31	0	0~58	11.11	
Established outcome measures only	7	0.18	0.04 to 0.32	0	0~58	9.80	
Studies of CBM combined with another intervention^d^	5	0.19	0.04 to 0.34	0	0~64	9.43	
*Subgroup analysis*^e^							
Addiction type Alcohol	5	0.18	0.03 to 0.33	0	0~64	9.80	0.933
Smoking	2	0.16	-0.23 to 0.56	0	N/A^i^	11.11	
Sample type Consumers	3	0.09	-0.28 to 0.46	0	0~73	20	0.608
Patients	4	0.19	0.04 to 0.34	0	0~68	9.43	
Bias targeted Approach	3	0.20	0.04 to 0.36	0	0~73	8.93	0.565
Attentional	4	0.10	-0.20 to 0.40	0	0~68	17.86	

Note.

^a^ All results are reported with Hedges *g*, using a random effects model

^b^ The *p* levels in this column indicate whether the difference between the ESs in the subgroups is significant (significant results are marked with italic)

^c^ Attwood et al., 2008; Field et al., 2005; Houben et al., 2011; Houben et al., 2012; WiersRW et al., 2010; Woud et al., 2015

^d^ Begh et al., 2015; Lopes et al., 2014; Schoenmakers et al., 2010; Wiers et al., 2011

^e^ Subgroup analysis were conducted using a mixed effects model.

^f^ One study (Boendermarker et al. 2015 Study 1) gave participants a choice between home and laboratory delivery

^g^ One study (Woud et al., 2015) used a different type of CBM (CBM for interpretation bias)

^h^ Attwood et al., 2008; Field et al., 2005; WiersRW et al., 2010; Woud et al., 2015

^i^ Confidence intervals around *I*^*2*^ cannot be calculated if there are fewer than 3 groups

^j^ The subgroups targeting inhibition and respectively interpretation bias only had one study

n = number of trials; NNT = numbers needed to treat; N/A not available. Underlined NNT values indicate negative ES values (the direction of the effect favored the control group)

**Fig 3:**
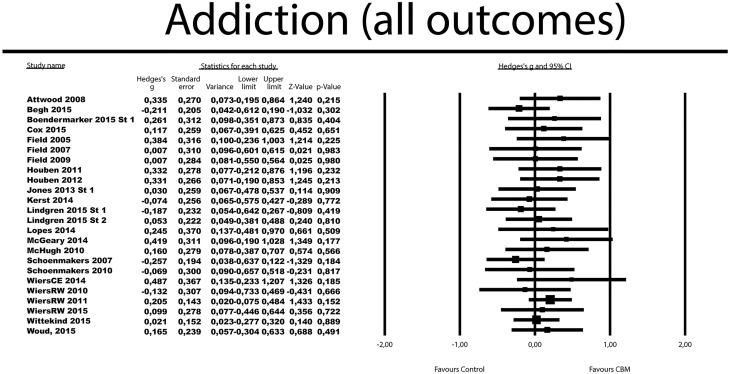
Standardized effect sizes of CBM interventions for addiction (all measures).

Fig 3 displays the forest plot of the standardized effect sizes of CBM interventions.

In four studies, more versions of CBM were included, which meant that multiple comparisons from these studies were not independent from each other. Consequently, their independent use in the same analyses could have affected the pooled ES through an artificial reduction of heterogeneity. We conducted sensitivity analyses by including only one ES per study (first the one with the largest ES, then the one with the lowest) to examine these possible effects. The resulting ESs as well corresponding heterogeneity were very close to the ones found in the overall analysis, with the difference that the ES for the analysis including the comparison most favorable for CBM from each study was small, but significant (*g* = 0.11, 95% CI 0.009 to 0.20).

The results were comparable when looking at alcohol and smoking dependence separately. For *alcohol* outcomes, 17 trials resulted into a non-significant *g* of 0.10 (95% CI -0.01 to 0.22), with zero heterogeneity (*I*^*2*^ = 0%, 95% CI: 0–45). For *smoking*, 7 RCTs aggregated in a non-significant *g* of 0.02 (95% CI -0.15 to 0.20), with zero heterogeneity (*I*^*2*^ = 0%; 95% CI: 0–58).

### Posttest results- Craving

Eighteen trials resulted in a non-significant *g* of 0.05 (95% CI -0.06 to 0.16), with zero heterogeneity (*I*^*2*^ = 0%) but a wider 95% CI (0–44). Results were similar when considering only one comparison for the studies with more comparisons, for established outcome measures only, when excluding comparisons with interventions aimed to increase bias, and for alcohol and smoking outcomes taken separately (Table 2).

### Posttest results- Cognitive bias (all measures)

Nineteen RCTs resulted in a significant *g* of 0.60 (95% CI 0.39 to 0.79) (Table 3). Heterogeneity was high (I^2^ = 64%; 95% CI: 36–77). Removal of 2 outliers reduced the ES to a *g* of 0.46 (95% CI 0.30 to 0.62), with heterogeneity remaining small but with a large 95% CI.

**Table 3:** Effects of CBM interventions, compared to control, at posttest, for bias outcomes^a^.

Variable	n_comp_	*g*	95% CI	*I*^*2*^	I^2^ 95% CI	p^b^
**Bias (all measures)**	19	0.60	0.39 to 0.79	64	36~77	
Outliers excluded^c^	17	0.46	0.310 to 0.62	39	0~65	
Comparisons with increase bias interventions excluded^d^	13	0.46	0.25 to 0.68	58	7~76	
*Subgroup analysis*^e^						
Addiction type Alcohol	14	0.59	0.37 to 0.80	59	12~76	0.927
Smoking	5	0.61	0.08 to 1.15	79	29–89	
Sample type Consumers	15	0.64	0.39 to 0.89	69	40~80	0.324
Patients	4	0.44	0.12 to 0.75	42	0~80	
Delivery setting^f^ Laboratory	13	0.70	0.42 to 0.98	71	43~82	0.213
Clinic	4	0.44	0.12 to 0.75	42	0~80	
Bias targeted^g^ Approach	5	0.46	0.12 to 0.81	63	0~84	0.677
Attentional	9	0.65	0.32 to 0.98	68	18~82	
Inhibition	4	0.70	0.13 to 1.27	76	0~89	
Number of sessions: Single	9	0.86	0.53 to 1.18	68	17~82	0.007
Multiple	10	0.35	0.16 to 0.53	28	0~65	

Note.

^a^ All results are reported with Hedges ***g*,** using a random effects model

^b^ The *p* levels in this column indicate whether the difference between the ESs in the subgroups is significant (significant results are marked with italic)

^c^ Outliers were defined as studies in the 95% CI was outside the 95% CI of the pooled studies. (Above the 95% CI: Attwood et al., 2008; Jones et al., 2013)

^d^ Attwood et al., 2008; Field et al., 2005; Houben et al., 2011; Houben et al., 2012; Wiers RW et al., 2010; Woud et al., 2015

^e^ Subgroup analysis were conducted using a mixed effects model.

^f^ One study employed delivery at home (Kerst and al., 2014) and another (Boendermarker et al. 2015 Study 1) gave participants a choice between home and laboratory delivery

^g^ One study (Woud et al., 2015) used a different type of CBM (CBM for interpretation bias)

n = number of trials

### Follow-up results- Addiction (all outcomes)

Seven trials reported follow-up outcomes, resulting in a small but significant *g* of 0.18 (95% CI 0.03 to 0.32), with zero heterogeneity (*I*^*2*^ = 0%). Result were significant for alcohol related addiction outcomes considered separately, *g* = 0.18, (95% CI 0.03 to 0.33), but not for smoking; *g* = 0.16, (95% CI -0.24 to 0.56). Due to lack of data, we did not calculate ESs for craving and bias. All follow-up trials involved multiple session CBM. While the studies had variable follow-up duration, the mean follow-up time frame was 6 months.

### Subgroup and meta-regression analysis

Subgroup analyses (Tables 2 and 3) revealed no significant between groups differences for addiction type, sample type, delivery setting, bias targeted. For addiction and craving outcomes at post-test, ESs were non-significant in any of these subgroups. For bias outcomes, ES were significant and highly heterogeneous in all subgroups. For number of sessions coded categorically (single versus multiple), there were no significant differences for addiction and craving outcomes, but for bias outcomes, studies with using a single session resulted in larger ESs than studies using multiple sessions.

**Fig 4:**
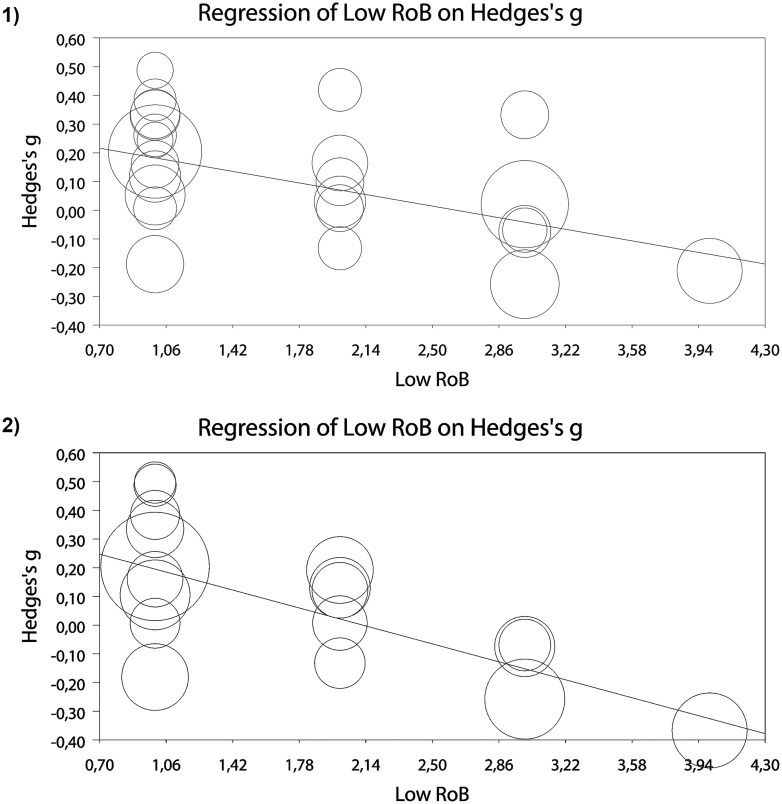
Meta-regression analyses for the effects of low risk of bias score on effects sizes for 1) addiction (all measures), and 2) craving.

Meta-regression analysis indicated a significant negative relationship between the low RoB score (number of criteria with low RoB) and ESs for addiction, slope b = -0.11, 95% CI -0.21 to—0.01; p = 0.026, and craving, slope b = -0.17, 95% CI -0.29 to—0.06; p = 0.001, at posttest (Fig 4). The relationship was not significant for bias outcomes.

However, since only 3 studies met more than 3 criteria for low RoB, we ran the analysis again excluding these studies. The relationship between risk of bias score and ES for addiction (all outcomes), slope b = -0.12, 95% CI -0.25 to 0.004; p = 0.059, remained borderline significant, while for craving, slope b = -0.17, 95% CI -0.33 to -0.002; p = 0.046, it was still significant. There was no significant association between number of sessions and ESs for addiction, craving or bias outcomes.

We also examined if the effects on cognitive bias were associated with the effects on addiction outcomes. To this purpose, we ran a meta-regression analysis using computed ES for cognitive bias as predictor and ESs on addiction (all measures) as dependent variable. The relationship was not significant, neither in the analysis including all 19 trials that had data for cognitive bias measures, slope b = 0.18, 95% CI -0.07 to 0.44, nor in the analysis excluding two studies that were identified as outliers, slope b = 0.29, 95% CI -0.09 to 0.63.

### Publication bias

Inspection of the funnel plot and the Duval-Tweedie trim and fill procedure documented some publication bias for addiction (all measures), craving and bias at posttest. For addiction, after adjusting for missing studies (n = 4), the ES decreased from a non-significant *g* of 0.07 to a non-significant *g* of 0.04 (95% CI -0.05 to 0.14). Adjusting for missing studies (n = 2) led to a non-significant *g* of 0.03, 95% CI -0.08 to 0.14, for craving. For bias, four studies were imputed, leading to a smaller, but significant *g* of 0.40, 95% CI 0.17 to 0.64. Egger’s test was not significant for any of these outcome categories (*p*> 0.05). For addiction outcomes at follow-up, the Duval-Tweedie trim and fill procedure did not impute any studies, and Egger’s test was not significant (*p*> 0.05).

## Discussion

CBM interventions have been viewed as holding particular promise for addiction problems, particularly due to their assumed capacity of acting upon automatic, impulsive processes, which are considered less amendable through traditional psychological interventions. While previous narrative reviews were generally positive, with one critical exception [11], our meta-analysis found no clinical benefits for these interventions at post-test, neither for all addiction outcomes, nor for craving. Results remained remarkably similar when we looked at alcohol or smoking-related outcomes separately, although CBM interventions have been viewed as especially effective for alcohol problems [9]. Subgroup analysis revealed a consistent pattern of results, contradicting recent claims about the potential superior effectiveness of multiple session CBM or when administered in the participant’s natural environment [11]. In fact, there was no indication that CBM might prove more efficient for certain types of applications or participants. Trials where CBM was administered in addition to another active intervention (e.g., cognitive behavioral therapy) were limited in number and also resulted in non-significant, close to zero, effects for addiction and craving. This contradicts recent claims that CBM would be more effective as an add-on to other interventions [27]. We did find a small, significant effect of CBM interventions at follow-up. However, we should be cautious in interpreting this result, as it was based on solely 7 trials, which according to our power analysis was insufficient, and all follow-ups were naturalistic making it difficult to attribute effects to interventions. This small number was even more problematic given there was a lot of variability across these studies regarding the duration of follow-up, sometimes even within the same study, or the outcome measures used. Moreover, a significant part of these trials did not conduct intent-to-treat analyses, rendering the results vulnerable to selective attrition. Consequently, it is possible this small effect might also be due to drop-out before follow-up, the passing of time, or supplementary interventions being accessed by participants.

We did, however, find an opposite pattern of results for cognitive bias outcomes: ESs were consistently large and significant and characterized by high heterogeneity, and this pattern was maintained across all subgroups. The only exception was that number of sessions had a small, but significant, negative association with ESs for bias: more sessions of CBM were related to a decrease in the magnitude of the effects on bias.

We found similar results (small and non-significant effects on symptom outcomes, but considerable moderate to large effects on cognitive bias) in a meta-analysis of CBM interventions in children and adolescents [7]. The consistency of this pattern for CBM interventions affords itself to various interpretations. Firstly, it could be that these interventions have a genuine effect on bias, but this does not translate into effects on symptoms (with the potential exception of follow-up measurements, which are arguably more important than post-test ones). Indeed, bias is often accepted as a surrogate outcome for clinical benefits in CBM trials, but this substitution could be unfounded. In support of this conjecture, our meta-regression results showed a non-significant relationship between ESs for cognitive bias and ESs for addiction outcomes. As results for bias were marked by high heterogeneity, this also indicates that some studies resulted in positive outcomes for bias and others did not. Alternatively, it could also be that more time may be needed for change of bias to be expressed in symptom change. While we did find a very small but significant effect for follow-up addiction outcomes, this was based on only 7 trials, and there were many differences as to the follow-up duration (4/6 trials had multiple follow-up points), and no control as to what other interventions participants accessed during that period. Importantly, in almost all these trials (5/7), participants also received another intervention along with CBM, making it difficult to attribute beneficial effects to CBM. All but one of these 7 trials did not employ any measures of addressing missing data, rendering them vulnerable to the effects of attrition bias. Most importantly however, trials with follow-up outcomes are few and come mostly from the same research group. Independent replication through methodologically sound trials done by independent researchers would be needed before ascertaining whether CBM might indeed be effective for addiction relevant outcomes at follow-up.

Finally, another explanation involves demand characteristics: as the task used for measuring bias and respectively for conducting intervention is generally the same (e.g., the visual dot probe or the approach-avoidance task), participants might simply be “getting better” at performing it. This also dovetails with the peculiar result from our subgroup analyses, where multiple sessions of CBM were associated with smaller ESs for cognitive bias than applications with just one session; as well as with the fact that in 7 out of the 14 trials that explicitly reported measuring contingency awareness, more than 50% of the participants (i.e., above chance) guessed the purpose of the task. Whatever the explanation, our results found little indication that positive effects on measures of cognitive bias translate to clinically relevant effects on addiction outcomes, with the potential exception of follow-up measurements. Thus, our results also contradict recent claims that the effectiveness of CBM interventions should only be evaluated in studies that successfully modify these biases [28].

To this point, we underscore that perhaps one of the most important problem our meta-analysis revealed was the problematic (i.e., high or uncertain) risk of bias in trials of CBM for addiction. Only 3 trials had low risk of bias for 3 or more of the 5 criteria we evaluated and most studies did not report sufficient information to permit assessment of bias. More strikingly, we showed a consistent pattern across addiction and craving outcomes of ESs decreasing in inverse relationship with the study’s global score for low risk of bias (i.e., computed by giving a point for each of the criteria considered on which the study could be rated as having low RoB). Simply put, studies that had low risk of bias on more criteria seemed to consistently result in *smaller* ESs. This pattern remained borderline significant for addiction (all outcomes) and significant for craving when the studies with low RoB on 3 or more of the criteria considered were excluded. Moreover, there was consistent evidence of publication bias across all outcome categories. Overall, trials of CBM for addiction have such a high or uncertain risk of bias on most of the criteria we analyzed, that one might question whether any reliable conclusion about the efficiency of these interventions could be extracted from them.

### Limitations

There are some limitations to our meta-analysis. One important one was small statistical power, given by the reduced number of trials, particularly in comparison to the different versions of CBM tasks tested, and this might have prevented us from evidencing smaller magnitude effects. Nonetheless, it is debatable whether such small effects could have any clinical importance. Another problem was the consistently moderate or large confidence intervals for *I*^*2*^, even when *I*^*2*^ was 0, indicating a heterogeneity was probably present, which most likely affected pooled ESs [22]. Related to this, we note that most studies used a wide range of outcome measurements and indices, focusing on various aspects of substance consumption or dependence. We tried to alleviate this problem by conducting separate analysis looking only at established, validated outcome measures and found similar results. Also, half of the trials were single sessions applications, and while we did not find any differences between these and the trials with more sessions, it could be argued that one session of CBM is unlikely to have therapeutic effects and such trials are less relevant in determining the efficiency of these interventions. Finally, most trials were focused on frequent or excessive consumers of a substance and not on diagnosed addiction patients. While we analyzed patients separately and found no evidence of an effect of CBM in this category, the number of trials was small and most of them came from the same research group. Consequently, we cannot exclude the possibility that more independent trials on dependent patients might result into a different pattern of results.

### Future directions

In a singular critical review of CBM interventions for relapse prevention in addiction [10], after pointing to methodological flaws and increasing negative results, the authors nonetheless end on an optimistic tone, underscoring “we are at beginning of a journey, rather than at the end” (p.49). Our results offer some possible suggestions for how this journey could continue. Steps should be taken to minimize trial risk of bias (or to make it less uncertain) and demand characteristics. Successful modification of cognitive bias, which authors of trials frequently interpret as an accomplishment of the intervention, does not seem to be related to symptom improvement. Consequently, trials with actual measures of symptom outcomes would be needed to gauge actual therapeutic benefits. There was no indication that certain applications of CBM would be more beneficial than others or that certain types of addiction problems would respond to these interventions better than to others. Therefore, constantly testing new versions of CBM tasks and procedures is most likely unnecessary and the development of a unified protocol to be used across more trials might be more suitable. The only area in which CBM appeared to have an effect on addiction outcomes was at follow-up; however, the small number of trials, high risk of attrition bias and their great variability render this result tentative. Future trials with similar follow-up durations, testing similar CBM interventions and accompanied by careful reporting of additional treatments participants might undergo during follow-up could further clarify whether this effect remains consistent.

## Supporting Information

S1 Fig Risk of bias summary: review authors' judgments about each risk of bias item for each included study. (PNG)

S1 File Search string for Pubmed. (DOCX)

S2 File List of studies included in the meta-analysis. (DOCX)

S3 File PRISMA Checklist. (DOCX)
