# Supplementary figures and images for: The Effectiveness of Cognitive Bias Modification Interventions for Substance Addictions: A Meta-Analysis
Source: PLoS One. 2016 Sep 9;11(9):e0162226. doi: 10.1371/journal.pone.0162226 (PMC5017662; doi:10.1371/journal.pone.0162226)

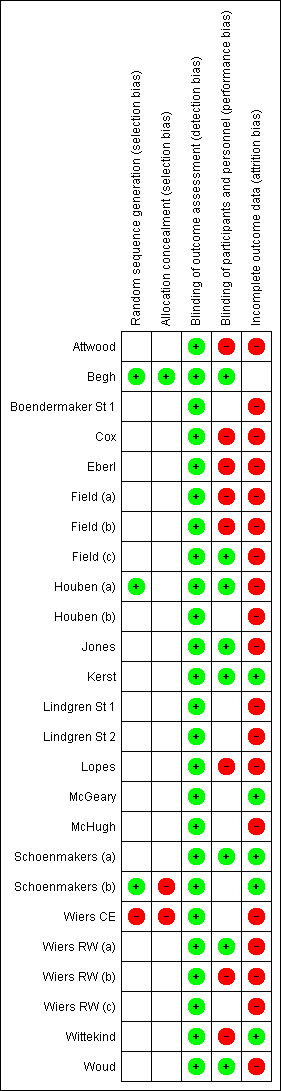

Supplement: S1 Fig — (PNG) [file pone.0162226.s001.png]
