# Supplementary material for: The Effectiveness of Cognitive Bias Modification Interventions for Substance Addictions: A Meta-Analysis
Source: PLoS One. 2016 Sep 9;11(9):e0162226. doi: 10.1371/journal.pone.0162226 (PMC5017662; doi:10.1371/journal.pone.0162226)
Supplement: S1 File — (DOCX) [file pone.0162226.s002.docx]

**S1 File. Search string for Pubmed**

PUBMED: (((((("cognitive bias"[Title/Abstract]) OR ("attention*"[Title/Abstract] AND "bias"[Title/Abstract]) OR ("interpret*"[Title/Abstract] AND "bias"[Title/Abstract]) OR ("approach" [Title/Abstract] NEAR "avoidance" [Title/Abstract])) AND (("modification"[Title/Abstract] OR "training"[Title/Abstract] OR "practice"[Title/Abstract] OR "task"[Title/Abstract])) AND ("addiction"[Title/Abstract] OR "dependen*"[Title/Abstract] OR "alcohol"[Title/Abstract] OR "drinking"[Title/Abstract] OR "tobacco"[Title/Abstract] OR "nicotine"[Title/Abstract] OR "smoking"[Title/Abstract] OR "drug"[Title/Abstract] OR "cannabis"[Title/Abstract] OR "marijuana"[Title/Abstract] OR "cocaine"[Title/Abstract] OR "heroin"[Title/Abstract] OR "opiates"[Title/Abstract] OR "amphetamine"[Title/Abstract] OR "substance *use"[Title/Abstract])))))

Date: 10^th^ of March

Hits: 183
