# Supplementary material for: The Effectiveness of Cognitive Bias Modification Interventions for Substance Addictions: A Meta-Analysis
Source: PLoS One. 2016 Sep 9;11(9):e0162226. doi: 10.1371/journal.pone.0162226 (PMC5017662; doi:10.1371/journal.pone.0162226)
Supplement: S2 File — (DOCX) [file pone.0162226.s003.docx]

**S2 File. List of studies included in the meta-analysis.**

1.

Attwood AS, O’Sullivan H, Leonards U, Mackintosh B, Munafò MR. Attentional bias training and cue reactivity in cigarette smokers. Addiction. 2008;103: 1875–1882. doi:10.1111/j.1360-0443.2008.02335.x

2.

Begh R, Munafò MR, Shiffman S, Ferguson SG, Nichols L, Mohammed MA, et al. Lack of attentional retraining effects in cigarette smokers attempting cessation: A proof of concept double-blind randomised controlled trial. Drug Alcohol Depend. 2015;149: 158–165. doi:10.1016/j.drugalcdep.2015.01.041

3.

Boendermaker WJ, Boffo M, Wiers RW. Exploring Elements of Fun to Motivate Youth to Do Cognitive Bias Modification. Games Health J. 2015;4: 434–443. doi:10.1089/g4h.2015.0053

4.

Cox WM, Fadardi JS, Hosier SG, Pothos EM. Differential effects and temporal course of attentional and motivational training on excessive drinking. Exp Clin Psychopharmacol. 2015;23: 445–454. doi:10.1037/pha0000038

5.

Eberl C, Wiers RW, Pawelczack S, Rinck M, Becker ES, Lindenmeyer J. Approach bias modification in alcohol dependence: do clinical effects replicate and for whom does it work best? Dev Cogn Neurosci. 2013;4: 38–51. doi:10.1016/j.dcn.2012.11.002

6.

Field M, Duka T, Eastwood B, Child R, Santarcangelo M, Gayton M. Experimental manipulation of attentional biases in heavy drinkers: do the effects generalise? Psychopharmacology (Berl). 2007;192: 593–608. doi:10.1007/s00213-007-0760-9

7.

Field M, Duka T, Tyler E, Schoenmakers T. Attentional bias modification in tobacco smokers. Nicotine Tob Res. 2009;11: 812–822. doi:10.1093/ntr/ntp067

8.

Field M, Eastwood B. Experimental manipulation of attentional bias increases the motivation to drink alcohol. Psychopharmacology (Berl). 2005;183: 350–357. doi:10.1007/s00213-005-0202-5

9.

Houben K, Havermans RC, Nederkoorn C, Jansen A. Beer à no-go: learning to stop responding to alcohol cues reduces alcohol intake via reduced affective associations rather than increased response inhibition. Addiction. 2012;107: 1280–1287. doi:10.1111/j.1360-0443.2012.03827.x

10.

Houben K, Nederkoorn C, Wiers RW, Jansen A. Resisting temptation: decreasing alcohol-related affect and drinking behavior by training response inhibition. Drug Alcohol Depend. 2011;116: 132–136. doi:10.1016/j.drugalcdep.2010.12.011

11.

Jones A, Field M. The effects of cue-specific inhibition training on alcohol consumption in heavy social drinkers. Exp Clin Psychopharmacol. 2013;21: 8–16. doi:10.1037/a0030683

12.

Kerst WF, Waters AJ. Attentional retraining administered in the field reduces smokers’ attentional bias and craving. Health Psychol. 2014;33: 1232–1240. doi:10.1037/a0035708

13.

Lindgren KP, Wiers RW, Teachman BA, Gasser ML, Westgate EC, Cousijn J, et al. Attempted Training of Alcohol Approach and Drinking Identity Associations in US Undergraduate Drinkers: Null Results from Two Studies. PLoS ONE. 2015;10: e0134642. doi:10.1371/journal.pone.0134642

14.

Lopes FM, Pires AV, Bizarro L. Attentional bias modification in smokers trying to quit: a longitudinal study about the effects of number of sessions. J Subst Abuse Treat. 2014;47: 50–57. doi:10.1016/j.jsat.2014.03.002

15.

McGeary JE, Meadows SP, Amir N, Gibb BE. Computer-delivered, home-based, attentional retraining reduces drinking behavior in heavy drinkers. Psychol Addict Behav. 2014;28: 559–562. doi:10.1037/a0036086

16.

McHugh RK, Murray HW, Hearon BA, Calkins AW, Otto MW. Attentional bias and craving in smokers: the impact of a single attentional training session. Nicotine Tob Res. 2010;12: 1261–1264. doi:10.1093/ntr/ntq171

17.

Schoenmakers T, Wiers RW, Jones BT, Bruce G, Jansen ATM. Attentional re-training decreases attentional bias in heavy drinkers without generalization. Addiction. 2007;102: 399–405. doi:10.1111/j.1360-0443.2006.01718.x

18.

Schoenmakers TM, de Bruin M, Lux IFM, Goertz AG, Van Kerkhof DHAT, Wiers RW. Clinical effectiveness of attentional bias modification training in abstinent alcoholic patients. Drug Alcohol Depend. 2010;109: 30–36. doi:10.1016/j.drugalcdep.2009.11.022

19.

Wiers CE, Stelzel C, Gladwin TE, Park SQ, Pawelczack S, Gawron CK, et al. Effects of cognitive bias modification training on neural alcohol cue reactivity in alcohol dependence. Am J Psychiatry. 2015;172: 335–343. doi:10.1176/appi.ajp.2014.13111495

20.

Wiers RW, Eberl C, Rinck M, Becker ES, Lindenmeyer J. Retraining automatic action tendencies changes alcoholic patients’ approach bias for alcohol and improves treatment outcome. Psychol Sci. 2011;22: 490–497. doi:10.1177/0956797611400615

21.

Wiers RW, Houben K, Fadardi JS, van Beek P, Rhemtulla M, Cox WM. Alcohol cognitive bias modification training for problem drinkers over the web. Addict Behav. 2015;40: 21–26. doi:10.1016/j.addbeh.2014.08.010

22.

Wiers RW, Rinck M, Kordts R, Houben K, Strack F. Retraining automatic action-tendencies to approach alcohol in hazardous drinkers. Addiction. 2010;105: 279–287. doi:10.1111/j.1360-0443.2009.02775.x

23.

Wittekind CE, Feist A, Schneider BC, Moritz S, Fritzsche A. The approach-avoidance task as an online intervention in cigarette smoking: a pilot study. J Behav Ther Exp Psychiatry. 2015;46: 115–120. doi:10.1016/j.jbtep.2014.08.006

24.

Woud ML, Hutschemaekers MHM, Rinck M, Becker ES. The manipulation of alcohol-related interpretation biases by means of Cognitive Bias Modification - Interpretation (CBM-I). J Behav Ther Exp Psychiatry. 2015; doi:10.1016/j.jbtep.2015.03.001
